# Maternal Diet-Induced Obesity Compromises Oxidative Stress Status and Angiogenesis in the Porcine Placenta by Upregulating Nox2 Expression

**DOI:** 10.1155/2019/2481592

**Published:** 2019-10-01

**Authors:** Chengjun Hu, Yunyu Yang, Jiaying Li, Hao Wang, Chuanhui Cheng, Linfang Yang, Qiqi Li, Jinping Deng, Zuman Liang, Yulong Yin, Zhengjun Xie, Chengquan Tan

**Affiliations:** ^1^Guangdong Provincial Key Laboratory of Animal Nutrition Control, Institute of Subtropical Animal Nutrition and Feed, College of Animal Science, South China Agricultural University, Guangzhou, Guangdong 510642, China; ^2^Guangdong Yihao Foodstuffs Co. Ltd., Guangzhou, Guangdong 510642, China; ^3^College of Animal Science and National Engineering Research Center for Breeding Swine Industry, South China Agricultural University, Guangzhou, Guangdong 510642, China; ^4^Guangdong Kondarl Agro-Pastoral Technology Co. Ltd., Dongguan, Guangdong 523142, China; ^5^National Engineering Laboratory for Pollution Control and Waste Utilization in Livestock and Poultry Production, Institute of Subtropical Agriculture, Chinese Academy of Sciences, Changsha, Hunan 410125, China; ^6^Shaoguan Jinpingguo Feeds Co. Ltd., Shaoguan, Guangdong 512027, China

## Abstract

Maternal obesity is associated with placental oxidative stress. However, the mechanism underlying this association remains poorly understood. In the present study, a gilt obesity model was developed by exposure to different energy diets and used to investigate the role of NADPH oxidase 2 (Nox2) in the placenta. Specifically, 99 gilts (Guangdong Small-ear Spotted pig) at day 60 of gestation were randomly assigned to one of the following three treatments: low-energy group (L, DE = 11.50 MJ/kg), medium-energy group (M, DE = 12.41 MJ/kg), and high-energy group (H, DE = 13.42 MJ/kg), with 11 replicate pens per treatment and 3 gilts per pen. At the start of the study, maternal body weight and backfat thickness were not significantly different in the three treatments. After the study, data indicated that the H group had higher body weight and backfat thickness gain for gilts during gestation and lower piglet birth weight compared with the other two groups. Additionally, the H group showed glucolipid metabolic disorders and increased triglyceride and nonesterified fatty acid contents in the placenta of gilts. Compared with the L group, the H group exhibited lower mitochondrial biogenesis and increased oxidative damage in the placenta. Importantly, increased mRNA expression and protein abundance of Nox2 were observed for the first time in H group placentae. Furthermore, compared with the L group, the H group showed a decrease in the density of placental vessels and the protein levels of vascular endothelial cadherin (VE-cadherin), vascular endothelial growth factor A (VEGF-A), and phosphorylation of vascular endothelial growth factor receptor 2 (p-VEGFR2) as well as the immunostaining intensity of platelet endothelial cell adhesion molecule-1 (CD31). Our findings suggest that maternal high-energy diet-induced obesity increases placental oxidative stress and decreases placental angiogenesis possibly through the upregulation of Nox2.

## 1. Introduction

Obesity has increased dramatically over the past few decades. From 1980 to 2013, the rate of women with a body mass index of ≥25 kg/m^2^ has increased from 29.8% to 38% [1]. Maternal obesity or overweight is one of the metabolic disorders for women at a reproductive age. Thus, it is not surprising that the number of obese or overweight women has increased during pregnancy over the past few decades. Maternal obesity or overweight is associated with several adverse outcomes, such as gestational diabetes mellitus, proteinuria preeclampsia, postpartum hemorrhage, fetal death, and low birth weight [2, 3], as well as poorer cognitive performance and increased risk of neurodevelopmental disorders for the offspring [4]. Additionally, offspring of obese mothers (BMI > 30) had an increased risk of hospital admission for a cardiovascular event [5]. However, the mechanisms underlying the development of these syndromes remain poorly understood.

Maternal obesity was associated with increased oxidative stress levels in the placenta [6], which is thought to be responsible for the occurrence of lower birth body weight [7]. However, the underlying mechanisms for this remain largely unknown. Placental blood vessels play an important role in exchanging nutrients, gases, and wastes between maternal and fetal circulations, suggesting that proper development of the placental vasculature is important for fetal growth. Studies have shown that oxidative stress is associated with vascular development. For instance, an increased amount of reactive oxygen species (ROS) has been found to induce autophagy, dysfunction, and apoptotic death of vascular endothelial cells [8, 9]. Therefore, it is possible that increased oxidative stress induced by maternal obesity may decrease the development of placental vasculature during pregnancy, thus contributing to lower birth body weight. Nicotinamide adenine dinucleotide phosphate (NADPH) oxidase 2 (Nox2) is the catalytic subunit of NADPH oxidase as well as a major source of ROS in the vasculature [10]. Deficiency in Nox2 was found to decrease ROS production and increase vascular densities in old animals [11]. In a pathological state, such as hypercholesterolemia, increased capillary density and decreased oxidative stress were observed in muscles of Nox2-deficient mice [12]. In addition, overproduction of ROS, upregulation of Nox2, and reduction of tube formation ability were observed in endothelial cells treated with angiotensin II [13]. Based on these lines of evidence available, Nox2 seems to be linked to oxidative stress and angiogenesis. Nevertheless, such role of Nox2 in the placenta has not been studied.

Gilts are one of the most commonly used animals in biomedical studies on human pregnancy due to their similarity in physiology [14–16]. Therefore, gilts with high backfat thickness induced by high-energy diet were used as an obesity model in the present study. To our knowledge, this is the first study to investigate the effects of maternal obesity on Nox2 expression in the placenta, and results showed that increased placental Nox2 expression was likely to induce oxidative stress and impair angiogenesis in the porcine placenta.

## 2. Materials and Methods

### 2.1. Animals and Experimental Treatments

**Figure 1:**
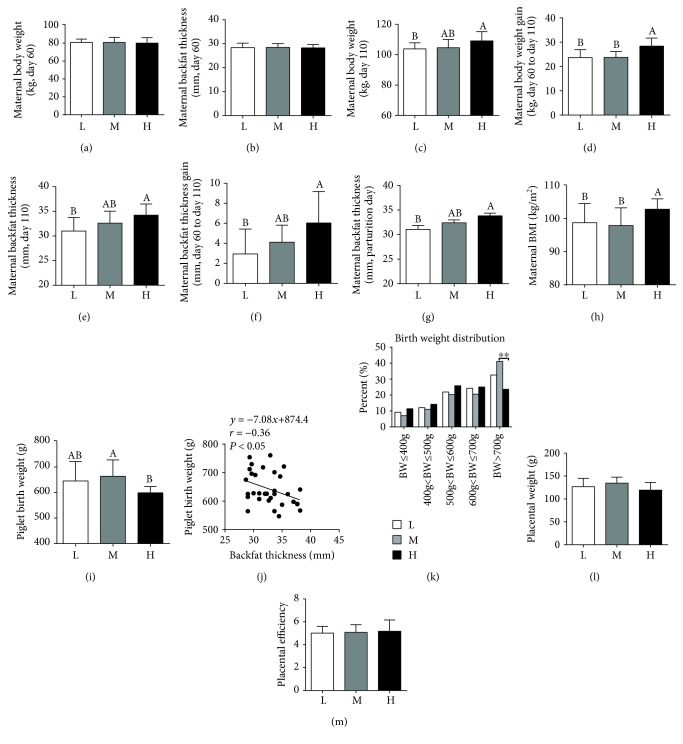
Maternal high-energy feeding increased maternal body weight and backfat thickness gain and decreased fetal birth weight. (a, b) Maternal body weight and backfat thickness at day 60 of gestation, respectively. (c, d) Maternal body weight and body weight gain at day 110 of gestation, respectively. (e, f) Maternal backfat thickness and backfat thickness gain at day 110 of gestation. (g) Maternal backfat thickness at parturition day. (h) Maternal body mass index (BMI) of gilts at day 110 of gestation. (i) Piglet birth weight. (j) Relationship between maternal backfat thickness at day 110 of gestation and piglet birth weight. (k) Birth weight distribution of piglets. Data were analyzed by the chi-square test. (l, m) Placental weight and efficiency, respectively. L = lower-energy group, M = median-energy group, H = high-energy group. Values are mean ± SD (*n* = 11). Different letters indicate significant differences at *P* < 0.05.

A total of 99 gilts (Guangdong Small-ear Spotted pig) that had been artificially inseminated with pooled semen from Duroc boars were entered into the study on day 60 of gestation. The gilts were obtained from a farm located in Suixi County, Guangdong Province, China. The animals were randomly assigned to one of the following three treatments: low-energy group (L), medium-energy group (M), and high-energy group (H). There were 11 replicate pens per treatment, with 3 gilts per pen. The digestible energy of the diet in the L, M, and H groups was 11.50 MJ/kg, 12.41 MJ/kg, and 13.42 MJ/kg, respectively (supplemental [Supplementary-material supplementary-material-1]). The nutrient level for the low-energy diet met the requirement of the Chinese National Feeding Standard (2004) for gestating gilts. Maternal body weight (80.72 ± 5.16 kg, mean ± SEM) and backfat thickness (28.37 ± 1.58 mm, mean ± SEM) at the start of the study were not significantly different in the three treatments (Figures 1(a) and 1(b)). At days 60-85, 86-90, and 91-110 during gestation, each pen (3 gilts) was provided with 3.6, 4.5, and 7.5 kg of diet per day, respectively, and had free access to drinking water. On gestation day 110, gilts were transferred to individual farrowing crates (2.2 m × 1.5 m). The experimental design and procedure presented in this study are reviewed and approved by the Animal Care and Use Committee of the South China Agricultural University.

### 2.2. Data Collection and Sampling

After parturition, the number of total piglets born was recorded and the birth weights of newborn piglets were measured within 6 h after birth. Backfat thickness of gilts was measured at P_2_ position at days 60 and 110 of gestation and day 0 of lactation using A-mode ultrasonography (Renco LEAN-MEATER®, Minneapolis, MN, USA). Body weight of gilts was measured at days 60 and 110 of gestation. The crown-rump length (the length from the crown of its head to the base of its tail) of gilts at day 110 of gestation was measured for calculation of the body mass index (BMI) [15].

When the gilts farrowed, umbilical cords were tied with a short silk line and labeled with a numbered tag to match individual piglets with their placentae [17]. Additionally, each piglet was labeled with an ear marker. After the placentae were expelled and their weights were recorded, the placentae were collected and snap-frozen in liquid nitrogen (approximately 5 g of each placenta, 3 to 4 cm from the cord insertion point). All the placentae were separated from the endometrium [18], and 5-6 placentae were collected from each gilt. Placental efficiency was calculated by dividing the piglet weight by the placental weight [17]. All placental samples used for laboratory analysis were chosen from their corresponding piglets with average birth weight. Eight gilts (from different pens) with average backfat thickness were randomly selected for blood sampling. Blood samples were collected from the ear vein of the fasted gilts using 10 mL centrifuge tubes at parturition day [19] and centrifuged at 3,000 × g and 4°C for 15 min to recover the serum [20].

### 2.3. Biochemical Parameter

Triglyceride (TG), glucose, and nonesterified fatty acid (NEFA) in serum were determined using the commercial kits (A110-1-1, F006-1-1, and A042-2-1; Nanjing Jiancheng Bioengineering Institute, Nanjing, China) according to the manufacturer's instructions. Insulin in serum was determined with an ELISA kit (CSB-E06829p; Cusabio, Wuhan, China) according to the manufacturer's instructions. Homeostasis model assessment‐insulin resistance (HOMA‐IR) = [(fasting insulin, mIU/L) × (fasting glucose, mmol/L)]/22.5; homeostasis model assessment‐insulin sensitivity (HOMA‐IS) = 1/[(fasting insulin, mIU/L) × (fasting glucose, mmol/L)] [21]. TG, NEFA, malondialdehyde (MDA), protein carbonyl, 8-hydroxy-2′-deoxyguanosine urine (8-OHdG), and glutathione (GSH) in the placenta were determined using the respective commercial kits (A110-1-1, A042-2-1, A003-1-2, A087-1-2, H165, and A006-2-1; Nanjing Jiancheng Bioengineering Institute, Nanjing, China). Placental reactive oxygen species (ROS) production was measured using 2′,7′-dichlorofluorescein diacetate (DCFH-DA) according to the manufacturer's protocol (E004; Nanjing Jiancheng Bioengineering Institute, Nanjing, China) as described previously [22].

### 2.4. Mitochondrial 4-Hydroxynonena (4-HNE)

The placental mitochondrial proteins were extracted using the Cytoplasmic and Mitochondrial Protein Extraction kit (C500051-0050; Sangon Biotech Co. Ltd., Shanghai, China). The protein concentration was determined using the BCA Protein Assay kit (P0012S; Beyotime, Shanghai, China). The 4-hydroxynonena was determined using a commercially available Elisa kit (RJ-25681; Shanghai Renjie Biotech Co. Ltd., Shanghai, China). All the above procedures were performed according to the manufacturer's instructions.

### 2.5. Adenosine Triphosphate (ATP), Nicotinamide Adenine Dinucleotide Reduced (NADH), and Nicotinamide Adenine Dinucleotide (NAD^+^) Levels

ATP, NAD^+^, and NADH levels in the placenta were determined using commercial kits (S0026, S0175, and S0175; Beyotime, Beijing, China) according to the manufacturer's instructions.

### 2.6. Citrate Synthase, Complex I, and III Activity

The citrate synthase activity was determined using a commercial kit (A108-2-1; Nanjing Jiancheng Bioengineering Institute, Nanjing, China). NADH ubiquinone oxidoreductase (complex I) and ubiquinol cytochrome reductase (complex III) activities were assessed spectrophotometrically using commercial kits (FHTA-2-Y, FHTC-1-Y; Cominbio Co., Suzhou, China). All the above tests were performed according to the manufacturer's instructions.

### 2.7. Mitochondrial DNA (mtDNA) Copy Number

Total genomic DNA was isolated from the placenta using the QIAamp DNA Mini Kit (51304; Qiagen, USA). The mitochondrial DNA copy number was determined using real-time PCR, using primers for mitochondrial cytochrome b (Cytb), and normalized to genomic DNA by amplification of the 18S rRNA as previously described [23].

### 2.8. Placental Oil Red O Staining and Vascular Density Determination

Placental tissues fixed in 4% paraformaldehyde were paraffin-embedded and sectioned at 5 *μ*m thickness [24], followed by staining with hematoxylin-eosin (H&E) and Oil red O. The area occupied by placental tissues was traced, and the placental vessels in these areas were also traced using a projecting microscope (Olympus CX41, Japan). Placental vascular areas were then quantified via image analysis and evaluated for the relative number of placental vessels per unit tissue area [25].

### 2.9. Immunohistochemistry

Immunohistochemistry for activating transcription factor 6 (*ATF*6) (GB11297; Servicebio, Wuhan, China) and glucose-regulated protein78 (*GRP*78) (GB11098; Servicebio, Wuhan, China) was performed on formalin-fixed paraffin-embedded tissue sections. Peroxidase-conjugated goat anti-rabbit IgG (GB23303; Servicebio, Wuhan, China) was used as the secondary antibody. The protocols of immunohistochemical staining have been described previously [26]. The cell nuclei were colored purple-blue, and positive products were tan or yellow. Three photographs were selected randomly for each slide and analyzed using ImageJ software (National Institutes of Health, Bethesda, MD).

### 2.10. Real-Time Quantitative PCR

Total RNA was isolated from the placental tissues using the TRIzol reagent (10296028; Invitrogen, Carlsbad, USA) [27]. The PrimeScript RT reagent kit (RR047A; Takara, Dalian, China) was used for reverse transcription of total RNA. Primers selected for PCR analyses were designed using Primer3 and are listed in supplemental [Supplementary-material supplementary-material-1]. Total reaction volume (20 *μ*L) comprised 2 *μ*L of cDNA template solution, 10 *μ*L of RealStar Green Fast Mixture (A304-01; GenStar, Beijing, China), 6.4 *μ*L of water, and 0.8 *μ*L of each primer. The relative expression of each target gene was determined using real-time (RT) PCR with an ABI QuantStudio™ 6 Flex system (Applied Biosystems, Carlsbad, CA). The RT-PCR program included a 10 min incubation at 95°C, followed by 40 cycles of denaturation for 15 s at 95°C and annealing and extension for 30 s at 60°C. Relative gene expression was expressed as a ratio of the target gene to the control gene using the formula 2^−(ΔΔCT)^ [28].

### 2.11. Western Blotting

Total proteins from the placentae were extracted by homogenizing and lysing in RIPA lysis buffer (P0013K; Beyotime, Shanghai, China) supplemented with phenylmethanesulfonyl fluoride (ST505; Beyotime, Shanghai, China), followed by separation with SDS-PAGE and blotting onto PVDF membranes. Blots were then incubated overnight at 4°C with the following primary antibodies: vascular endothelial growth factor A (VEGF-A) polyclonal antibody (19003-1-AP; Proteintech, USA, 1 : 1000 dilution), vascular endothelial-cadherin (VE-cadherin) polyclonal antibody (2500T; CST, USA, 1 : 1000 dilution), vascular endothelial growth factor receptor 2 (VEGFR2) polyclonal antibody (abs120032; Absin Bioscience Inc., Shanghai, China, 1 : 1000 dilution), phospho-VEGF Receptor 2 (Tyr1175) polyclonal antibody (2478; CST, USA, 1 : 1000 dilution), Nox2/gp91phox Polyclonal Antibody (abs124860; Absin Bioscience Inc., Shanghai, China, 1 : 1000 dilution), and rabbit monoclonal anti-NADPH oxidase 4 (Nox4) (ab109225; Abcam, USA, 1 : 1000 dilution). The density of bands was quantified using ImageJ software (National Institutes of Health, Bethesda, MD) and then normalized to *β*-actin (4970; CST, USA, 1 : 1000 dilution).

### 2.12. Immunofluorescence

Placental tissues fixed in 4% paraformaldehyde were paraffin-embedded and sectioned at 5 *μ*m thickness for von Willebrand factor (vWF) and platelet endothelial cell adhesion molecule-1 (CD31) immunofluorescence. Placentae were deparaffinized in xylene and rehydrated in grade alcohol. Placental tissues were dipped in citrate buffer (Servicebio, Wuhan, China) at 120°C in a pressure cooker for 5 min and washed 3 times in PBS (pH 7.4) for 5 min. Slides were then blocked with 3% bovine serum albumin for 30 min, followed by incubation with the vWF primary antibody (GB11020; Servicebio, Wuhan, China) and CD31 primary antibody (GB13063; Servicebio, Wuhan, China) diluted to 1 : 200 in PBS overnight at 4°C. Then, the slides were washed 3 times for 10 min in PBS (pH 7.4) followed by incubation for 30 min in a CY3 rabbit anti-goat antibody (GB21404; Servicebio, Wuhan, China) diluted to 1 : 300 in PBS. Next, slides were washed 3 times for 5 min in PBS and stained with 4′,6-diamidino-2-phenylindole (DAPI) solution in a dark room for 10 min. Finally, the slides were visualized under a fluorescent microscope (Nikon Eclipse C1, Tokyo, Japan). Fluorescence intensities were quantified using ImageJ software (National Institutes of Health, Bethesda, MD).

### 2.13. Statistical Analysis

The data of piglet birth weight, NAD^+^, NAD^+/^NADH, and complex I activities as well as the mRNA expression levels of nuclear respiratory factor 1 (*NRF-1*), *β*-subunit of the mitochondrial H^+^-ATP synthase (*β-F1-ATPase*), *Nox*4, and cytochrome c (*Cytc*) were nonnormally distributed and analyzed using the Kruskal-Wallis test. Placental ATP levels were analyzed using one-way ANOVA, followed by Tamhane's T2 test in SPSS 20.0 (SPPS Inc., Chicago, IL). Other variables (excluding the nonnormally distributed variables) were analyzed using one-way ANOVA, followed by Duncan's multiple-range test [29]. Data were presented as mean ± SD (standard deviation) from corresponding groups (*n* = 8-11). The statistics was performed based on sow replication. Piglet birth weight distribution was analyzed by the chi-square test. Differences between mean values were considered statistically significant at *P* < 0.05.

## 3. Results

### 3.1. Characteristics of Gilts and Piglets

Characteristics of the gilts are presented in Figure 1. Compared with the L group, maternal high-energy feeding increased maternal body weight (Figure 1(c)) and backfat thickness (Figure 1(e)) at day 110 of gestation, as well as body weight and backfat thickness gain (Figures 1(d) and 1(f)). Moreover, maternal BMI at day 110 of gestation was significantly higher in the H group than in the L or M group (Figure 1(h)).

Compared to the M group, the H group showed a significant decrease in piglet birth weight (Figure 1(i)), with a significant negative correlation observed between backfat thickness and piglet birth weight (Figure 1(j)). The percentage of piglets with a birth weight > 700 g was higher in the M group than in the H group (Figure 1(k)).

### 3.2. Metabolic Characterization of Gilts

**Figure 2:**
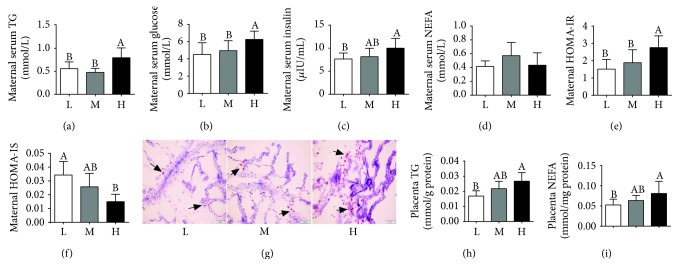
Metabolic characterization of gilts. Maternal serum TG (a), glucose (b), insulin (c), and NEFA (d). *n* = 8. (e) HOMA‐IR = [fasting glucose (mmol/L) × fasting insulin (*μ*U/mL)]/22.5. (f) HOMA‐IS = 1/[(fasting insulin, *μ*IU/L) × (fasting glucose, mmol/L)]. (g) Placental oil red O staining (×200 magnification, bar = 100 *μ*m). (h, i) Placental TG and NEFA levels, respectively. Values are mean ± SD (*n* = 11). Different letters indicate significant differences at *P* < 0.05.

As shown in Figure 2, compared to the L or M group, the H group had an obvious increase in the maternal serum TG and glucose levels (Figures 2(a) and 2(b)). An increase in the maternal serum insulin level was also observed in the H group relative to the L group (Figure 2(c)), whereas no significant differences were observed in the maternal serum NEFA levels among the 3 groups (Figure 2(d)). In addition, higher HOMA-IR and lower HOMA-IS were observed in the H group compared to the L group (Figures 2(e) and 2(f)).

Furthermore, placental TG and NEFA levels were higher in the H group than in the L group (Figures 2(g)–2(i)).

### 3.3. Oxidative Stress in the Placenta

**Figure 3:**
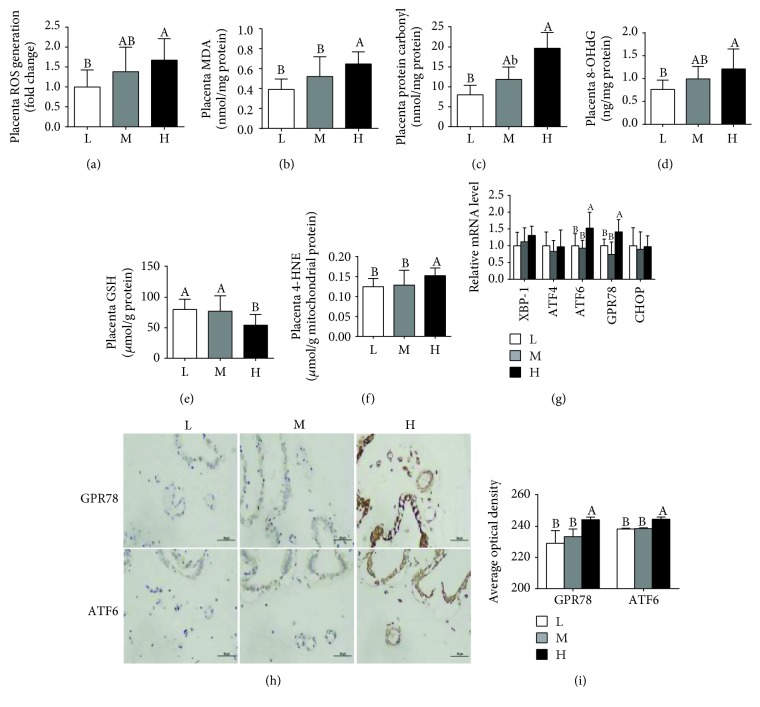
Maternal high-energy feeding increased the oxidative stress level in the placenta. Placental ROS (a), MDA (b), protein carbonyl (c), 8-OHdG (d), GSH (e), and mitochondrial 4-HNE (f) levels. (g) The mRNA expression level of the endoplasmic stress markers. (h) Immunohistochemistry for placental ATF6 and GRP78 (×400 magnification, bar = 50 *μ*m). (i) Average optical density of ATF6 and GRP78. Values are mean ± SD (*n* = 8-11). Different letters indicate significant differences at *P* < 0.05.

As shown in Figure 3, compared to the L group, the H group exhibited a notable increase in placental ROS, MDA, protein carbonyl, and 8-OHdG (Figures 3(a)–3(d)). When compared to the L or M group, the H group had a significant decrease in the placental GSH level (Figure 3(e)) and an increase in the mitochondrial 4-HNE level (Figure 3(f)). However, no obvious differences were observed in the ROS, MDA, and protein carbonyl levels between the L group and M group. The mRNA expression levels of the endoplasmic stress markers *GRP*78 and *ATF*6 were higher in the H group than in the L or M group (Figure 3(g)). Immunohistochemistry analysis also revealed higher expression levels of GPR78 and ATF6 in the H group than in the L or M group (Figures 3(h) and 3(i)).

### 3.4. Placental ATP Levels and Mitochondrial Biogenesis

**Figure 4:**
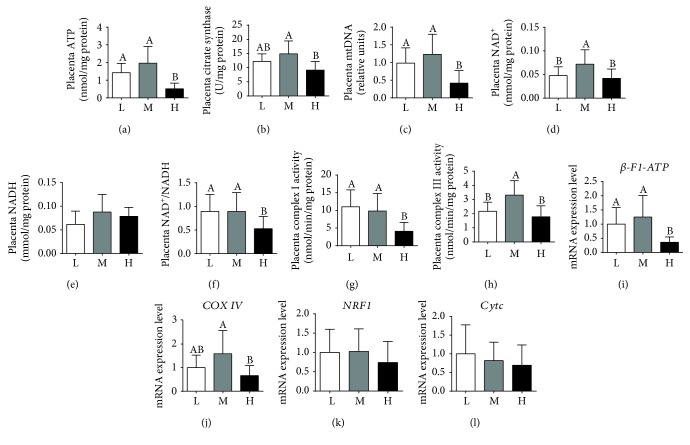
Maternal high-energy feeding decreased ATP levels and mitochondrial biogenesis in the placenta. ATP levels were normalized to the total protein level (a). Data were analyzed using one-way ANOVA, followed by Tamhane's T2 test. Mitochondrial biogenesis was estimated by citrate synthase activity (b), mitochondrial DNA (mtDNA) copy number (c), NAD^+^ (d), NADH (e), and the ratio of NAD^+^/NADH (f). Complex I (g) and complex III (h) activities were normalized to the total protein level. The relative mRNA expression levels of *β-F1-ATP* (i), *COXIV* (j), *NRF*1 (k), and *Cytc* (l) were measured by qPCR. Values are mean ± SD (*n* = 11). Different letters indicate significant differences at *P* < 0.05.

As shown in Figure 4, the H group was significantly lower than the L or M group in placental ATP levels (Figure 4(a)), the amount of mtDNA (Figure 4(c)), NAD^+^ (Figure 4(d)), and the ratio of NAD^+^/NADH (Figure 4(f)). The H group also showed a significant decrease in the citrate synthase activity when compared with the M group (Figure 4(b)). The complex I activity was lower in the H group than in the L or M group (Figure 4(g)), and the complex III activity was higher in the M group than in the L or H group (Figure 4(h)). Furthermore, the H group was lower than the M group in the mRNA expression levels of *β-F1-ATP* and cytochrome c oxidase IV (*COXIV*) (Figures 4(i) and 4(j)). Meanwhile, the mRNA expression level of *β-F1-ATP* was higher in the L group than in the H group (Figure 4(i)), whereas no obvious differences were observed among the 3 groups in the mRNA expression levels of *NRF-1* and *Cytc* (Figures 4(k) and 4(l)).

### 3.5. Nox2 and Nox4 mRNA and Protein Expression Levels in the Placenta

**Figure 5:**
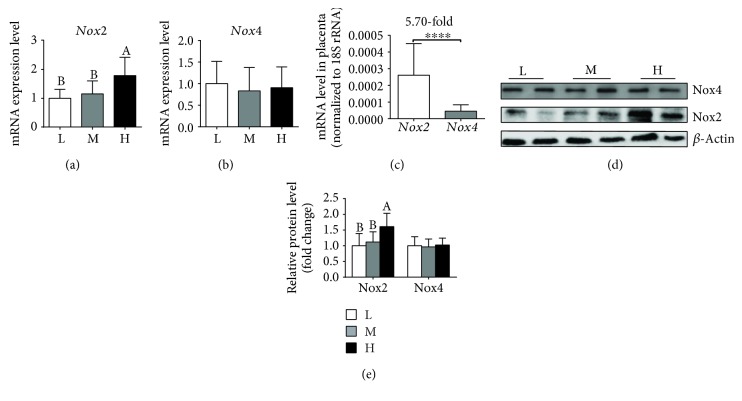
Maternal high-energy feeding increased Nox2 mRNA and protein expression in the placenta. The mRNA (a) and protein (d) expression levels of Nox2 were determined by qPCR and Western blot, respectively. Placental Nox4 mRNA (b) and protein (d) expression in the placenta. (c) Comparison of *Nox*2 and *Nox*4 mRNA expression level in the placenta. (e) Summarized data of the protein expression of Nox2 and Nox4. Values are mean ± SD (*n* = 11). Different letters indicate significant differences at *P* < 0.05.

As shown in Figure 5, the H group showed a significant increase over the L or M group in the mRNA (Figure 5(a)) and protein (Figure 5(d)) expression levels of Nox2, whereas no notable differences were observed between the L and M groups. Furthermore, the three groups exhibited no significant difference in the mRNA (Figure 5(b)) and protein (Figure 5(d)) expression levels of Nox4 in the placenta, with the mRNA level of *Nox*2 being obviously higher than that of *Nox*4 (Figure 5(c)).

### 3.6. Placental Vessel Density and Expression of the Key Genes Related to Angiogenesis

**Figure 6:**
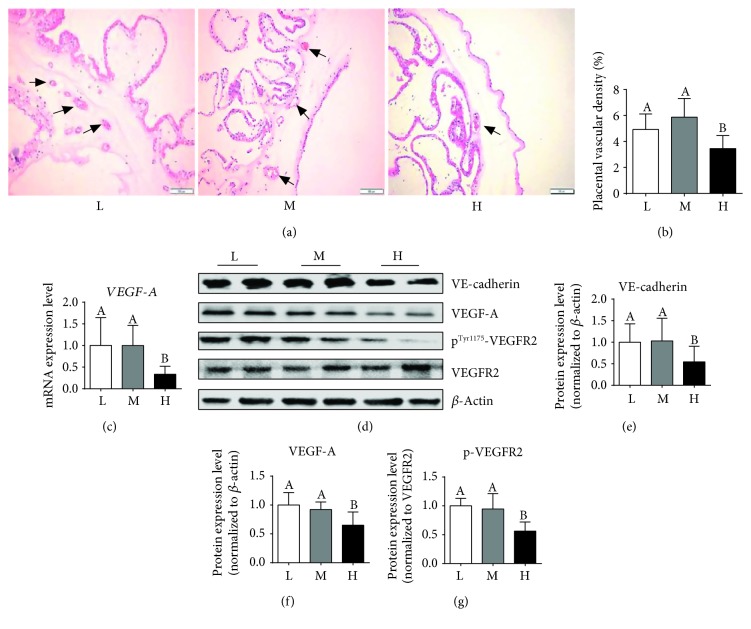
Maternal high-energy feeding decreased the placental angiogenesis. The vessels in placental tissues were stained using hematoxylin-eosin (H&E) (×200 magnification, bar = 100 *μ*m) (a), and the arrows indicate the placental vessels. Quantitative number of vessels per unit area of placental tissues (b). The mRNA (c) and protein (d) expression levels of VEGF-A were determined by qPCR and Western blot, respectively. Values are mean ± SD (*n* = 11). Different letters indicate significant differences at *P* < 0.05. (e–g) Summarized data of VE-cadherin, VEGF-A, and p-VEGFR2.

Figure 6(a) shows the distribution of vessels in the placental tissues as identified by H&E staining. Quantitative areas of vessels per unit area of placental tissues are presented in Figure 6(b), with a marked decrease in the H group. When compared with the L or M group, the H group also had a significant decrease in the mRNA expression (Figure 6(c)) and protein abundance of VEGF-A, VE-cadherin, and p-VEGFR2 (Figures 6(d)–6(g)).

**Figure 7:**
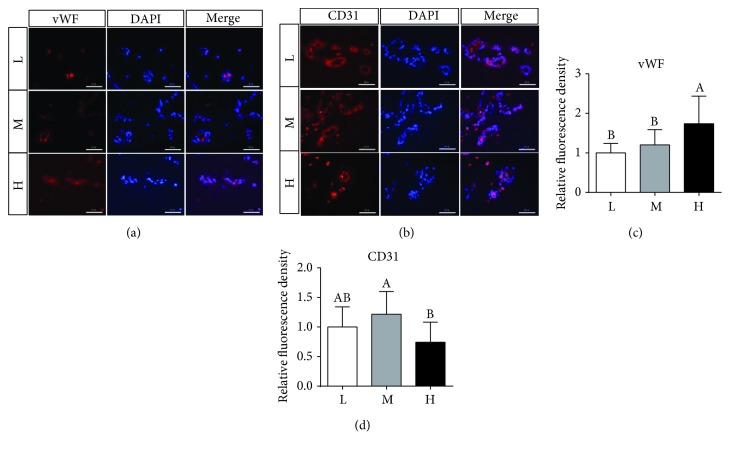
vWF (a) and CD31(b) immunofluorescence staining in the placenta (×400 magnification, bar = 50 *μ*m). Summarized data of vWF (c) and CD31 (d). Values are mean ± SD (*n* = 11). Different letters indicate significant differences at *P* < 0.05.

The vWF and CD31 immunofluorescent staining intensities were evaluated in placentae. When compared with the M group, the H group showed decreased immunostaining intensity of CD31 (Figures 7(b)–7(d)) and increased immunostaining intensity of vWF in the placentae (Figures 7(a)–7(c)). Furthermore, the H group was also obviously higher than the L group in the vWF immunostaining intensity.

## 4. Discussion

Maternal obesity increases the oxidative stress in the placenta [6]. Increased oxidative stress is obvious in the placenta with intrauterine growth restriction, suggesting that oxidative stress may contribute to the occurrence of lower birth weight [30, 31]. However, the molecular mechanism underlying this link remains unclear. In pigs, a high occurrence of intrauterine growth restriction is widely described for obese swine [32, 33]. Additionally, the pig has been reported as a better animal model for investigation of human obesity [34]. Therefore, gilts with obesity induced by diet were used to investigate the mechanism for the effects of maternal obesity on fetal growth and placental function. In the present study, gilts fed high-energy diet during gestation showed a marked increase in backfat thickness, BMI, and body weight at day 110 of gestation, as well as serum TG, glucose, and insulin at parturition day, indicating that the gilts were fatter in the H group than in the other groups. Oxidative stress is recognized as an imbalance between the production of free radicals and the capacity of antioxidant defenses to scavenge them. Our results confirmed that maternal obesity creates a state of increased oxidative stress within the placenta, as evidenced by the increased placental ROS level, which was in line with a previous study showing that maternal adiposity increased the placental ROS level [6]. Excessive ROS accumulation causes oxidative damage to lipids and protein primarily through lipid and protein peroxidation [35, 36]. The higher levels of MDA, protein carbonyl, and 4-HNE in placentae indicated greater lipid and protein peroxidation in the placentae of obese gilts, which was in line with the observed increase in ROS levels. Additionally, the H group also showed an increase in the mRNA and protein expression levels of endoplasmic reticulum stress markers GRP78 and ATF6. These results further confirmed that the placentae from obese gilts were subjected to higher oxidative stress. Increased oxidative stress in placentae was associated with the occurrence of intrauterine growth restriction [7], and the birth weight was lower in the H group in the present study. In addition, maternal obesity leads to the accumulation of excess fatty acids in the placenta, resulting in a lipotoxic placental environment. Lipotoxicity has the ability to influence placental function, suggesting that the accumulation of excess fatty acids may have a link with maternal obesity and placenta-related adverse pregnancy [37]. A previous study has reported that elevated free fatty acids were shown to stimulate ROS production [38]. Therefore, it is not surprising that excess ROS production was observed in the placenta of obese gilts.

ROS formation is the first step in the one-electron reduction of molecular oxygen. Mitochondrial complex I and complex III in the transport chain were considered the major ROS generation site, which causes less than 4% of oxygen to be reduced to superoxide anion and generate oxidative stress [39, 40]. Most of the free radical oxygen species are generated by complex I, and deficiency of mitochondrial complex I leads to increased production of superoxide radicals [41]. In the three groups, the placental complex I and III activities were lowest in the obese gilts, suggesting that mitochondria may be functionally impaired with increasing maternal obesity. ROS is also well documented to be produced through NADPH oxidase activation [38]. Nox2 and Nox4 are the main catalytic subunit and cytochrome subunit of the phagocyte NADPH oxidase [42, 43]. An important observation in this study is that, when compared with the L or M group, gilts fed high-energy diet (H group) showed an increase in the mRNA and protein levels of Nox2, but no obvious change in the Nox4 mRNA and protein level in the placenta. Moreover, the mRNA expression of Nox2 was higher than that of Nox4 in the placenta, suggesting that Nox2 is the main isoform of NADPH oxidase in the placenta. Inhibition or loss of Nox2 prevents oxidative stress and mitochondrial abnormality [44, 45]. Therefore, excess ROS production in the placenta of obese gilts may be partially attributed to the upregulation of NOX2 expression. Interestingly, decreased placental mitochondrial DNA copy number, ATP, the ratio of NAD^+^/NADH, and citrate synthase activity were also observed in obese gilts, indicating that maternal obesity induced by diet leads to abnormal mitochondrial biogenesis. Mitochondria are not only one of the main ROS generation sites but also a target of ROS attack [46]. Many lines of evidence have shown that mitochondrial dysfunction results from oxidative stress in the skeletal muscle, heart, and liver [47–49]. Therefore, we speculate that upregulated Nox2 levels lead to increased oxidative stress in the placenta, thus contributing to abnormal mitochondrial biogenesis [44]. However, the mechanism needs to be further elucidated.

The blood vessels within the placenta are responsible for exchanging nutrients between the mother and fetus. Aberrant angiogenesis in the placenta can lead to fetal growth restriction [50]. In the present study, placental vascular density was the lowest in obese gilts among the three groups, which was in line with Stuart et al. who reported that diet-induced obesity decreases placental vascularity in the mouse [51]. CD31 is a commonly used biomarker of the endothelial cell in small vessels [52]. VE-cadherin is the junction adhesion molecule uniquely expressed in endothelial cells and acts to promote angiogenesis [53]. The placental VE-cadherin protein abundance and CD31 immunofluorescence staining data further demonstrated that high-energy diet induces the reduction of vessels in the placenta. VEGF plays an important role in angiogenesis, and VEGFR2 is responsible for downstream VEGF signaling and the major signal transducer for angiogenesis [13]. Here, the protein levels of VEGF and p-VEGFR2 were remarkably decreased in the placenta of obese gilts. These data indicate that maternal obesity induced by high-energy diet can modulate placental angiogenesis by inhibiting the VEGF/VEGFR2 signaling pathway.

ROS serve as a double-edged sword in the vasculature. High levels of ROS are harmful to endothelial cells, whereas low levels of ROS can activate signaling pathways and promote angiogenesis [40, 54, 55]. vWF, a marker of vascular inflammation [56], increased in the placentae of obese gilts, which was consistent with the observed increase in ROS levels, suggesting that increased oxidative stress leads to vascular endothelial dysfunction [31]. Several studies have shown the involvement of Nox2 in the modulation of arterial tone and human atherosclerosis disease and the association of its deficiency with reduced atherosclerotic burden [57, 58], implying that Nox2 may play an important role in vascular damage. Furthermore, Nox2 may also exert an important function in angiogenesis. As reported previously, Nox2 deficiency reduces oxidative stress levels in ischemic tissues and increases vascular densities in old animals [11, 12]. Excess accumulation of ROS and a decrease in tube formation ability, coupled with Nox2 upregulation and p-VEGFR2/VEGFR2 reduction, were observed in an angiotensin II-induced oxidative stress model [13]. Moreover, Nox2-derived superoxide leads to vascular dysfunction in diet-induced obesity [59]. All these reports may help to explain the reasons for the increase in Nox2 protein level and the decrease in vascular density in the placentae of obese gilts. Based on these reports and the results in the present study, the decreased angiogenesis in the placentae of obese gilts may be attributed to the upregulation of Nox2 expression.

## 5. Conclusion

In this study, diet-induced obesity in the gilts was demonstrated to profoundly increase the placental oxidative stress level and decrease the mitochondrial biogenesis and vascular density that are essential for fetal growth, with the mechanisms involved in the upregulation of Nox2 in the placenta. These results contribute to a better understanding of the role of Nox2 in maternal obesity. Furthermore, the modulation of Nox2 oxidase activity in the placenta may have the potential as a novel therapeutic approach to reduce oxidative stress, restore angiogenesis, and improve fetal growth.

## Data Availability

The data used to support the findings of this study are available from the corresponding authors upon request.

## Supplementary Materials

Supplementary Materials Supplementary Table 1: composition and nutrient levels of the experimental diets. Supplemental Table 2: primers used for real-time PCR. ATF4/6: activating transcription factor 4/6; Cytc: cytochrome c; COX IV: cytochrome c oxidase IV; CHOP: C/EBP-homologous protein; GRP78: glucose-regulated protein78; *β*-F1-ATPase: *β*-subunit of the mitochondrial H^+^-ATP synthase; NRF1: nuclear respiratory factor 1; Nox2: NADPH oxidase 2; Nox4: NADPH oxidase 4; 18MT-CYB: mitochondrially encoded cytochrome b; VEGF-A: vascular endothelial growth factor A; XBP-1: X-box binding protein-1; S rRNA: 18S ribosomal RNA.
