# Supplementary material for: Maternal Diet-Induced Obesity Compromises Oxidative Stress Status and Angiogenesis in the Porcine Placenta by Upregulating Nox2 Expression
Source: Oxid Med Cell Longev. 2019 Oct 1;2019:2481592. doi: 10.1155/2019/2481592 (PMC6791269; doi:10.1155/2019/2481592)
Supplement: Supplementary Materials — Supplementary Table 1: composition and nutrient levels of the experimental diets. Supplemental Table 2: primers used for real-time PCR. ATF4/6: activating transcription factor 4/6; Cytc: cytochrome c; COX IV: cytochrome c oxidase IV; CHOP: C/EBP-homologous protein; GRP78: glucose-regulated protein78; β-F1-ATPase: β-subunit of the mitochondrial H+-ATP synthase; NRF1: nuclear respiratory factor 1; Nox2: NADPH oxidase 2; Nox4: NADPH oxidase 4; 18MT-CYB: mitochondrially encoded cytochrome b; VEGF-A: vascular endothelial growth factor A; XBP-1: X-box binding protein-1; S rRNA: 18S ribosomal RNA. [file 2481592.f1.docx]

Supplementary Table 1: Composition and nutrient levels of the experimental diets.

Supplemental Table 2. Primers used for real-time PCR. ATF4/6: activating transcription factor 4/6; Cytc: cytochrome c; COX IV: Cytochrome c oxidase IV; CHOP: C/EBP-homologous protein; GRP78: glucose-regulated protein78; β-F1-ATPase: β subunit of the mitochondrial H^+^-ATP synthase; NRF1: nuclear respiratory factor 1; Nox2: NADPH oxidase 2; Nox4: NADPH oxidase 4; 18MT-CYB: mitochondrially encoded cytochrome b; VEGF-A: Vascular endothelial growth factor A; XBP-1: X-box binding protein-1; S rRNA: 18S ribosomal RNA

Supplemental Table 1. Composition and nutrient levels of the experimental diets (as-fed basis) ^1^

| Items | L | M | H |
| --- | --- | --- | --- |
| Ingredient, % |  |  |  |
| Corn | 32.09 | 47.89 | 59.39 |
| Soybean meal | 10.00 | 12.80 | 14.20 |
| Barley | 13.00 | 13.00 | 0.00 |
| Wheat brain | 24.00 | 13.90 | 16.00 |
| [soybean](file:///D:\360%E5%AE%89%E5%85%A8%E6%B5%8F%E8%A7%88%E5%99%A8%E4%B8%8B%E8%BD%BD\Dict\7.5.2.0\resultui\dict\?keyword=soybean)[hull](file:///D:\360%E5%AE%89%E5%85%A8%E6%B5%8F%E8%A7%88%E5%99%A8%E4%B8%8B%E8%BD%BD\Dict\7.5.2.0\resultui\dict\?keyword=hull) | 16.30 | 7.80 | 2.80 |
| Soybean oil |  |  | 3.00 |
| Calcium carbonate | 0.50 | 0.50 | 0.50 |
| Premix^2^ | 4.11 | 4.11 | 4.11 |
| Total | 100.00 | 100.00 | 100.00 |
| Chemical composition^3^ |  |  |  |
| DE, MJ/kg | 11.50 | 12.41 | 13.42 |
| CP, % | 13.48 | 13.46 | 13.44 |
| EE, % | 2.86 | 2.86 | 5.99 |
| CF, % | 9.37 | 5.99 | 4.13 |
| Ca, % | 1.07 | 1.02 | 1.00 |
| P, % | 0.65 | 0.60 | 0.61 |
| Lys, % | 0.63 | 0.64 | 0.63 |
| Met+Cys, % | 0.57 | 0.53 | 0.52 |
| Trp, % | 0.16 | 0.16 | 0.16 |
| Thr, % | 0.52 | 0.52 | 0.53 |

^1^ L= lower energy group; M=median energy group; and H=high energy group.

^2^Premix provided for 1 kg of complete diet: Cu,75 mg; Fe,750 mg; Se, 0.20 mg; Zn, 750 mg; Mn, 187 mg; vitamin A, 150,000 IU; vitamin B_2_, 140 mg; vitamin D_3_, 25,000 IU; vitamin E, 72 IU; vitamin K_3_, 35 mg; vitamin B_2_, 140 mg; vitamin B_6,_ 70mg; calcium pantothenate, 350 mg; niacin, 500 mg; and vitamin B_12_, 0.4 mg.

^3^ Calculated value using values for feed ingredients from the Nutrient requirements of swine (2012).

**Supplemental Table 2.** Primers used for real-time PCR

| Genes | Primers | Primers Sequences (5^，^to 3^，^) | Size (bp) |
| --- | --- | --- | --- |
| ATF4 | Forward | AACATGGCCGAGATGAGCTTCC | 265 |
|  | Reverse | TCTCCACCATCCAGTCTGTCCC |  |
| ATF6 | Forward | CTCAGCTCATGGCTGTCCAA | 136 |
|  | Reverse | AATGTGTCTCCCCTTCTGCG |  |
| Cyt c | Forward | TAGAAAAGGGAGGCAAACACAAG | 154 |
|  | Reverse | GGATTCTCCAGGTACTCCATCAG |  |
| COX IV | Forward | CCAAGTGGGACTACGACAAGAAC | 131 |
|  | Reverse | CCTGCTCGTTTATTAGCACTGG |  |
| CHOP | Forward | TTAAGTGTGACAAGGAGAAGAAC | 207 |
|  | Reverse | CAAGGAAGGCAGAATAGAAGC |  |
| GRP78 | Forward | AGTCCCGCAGATTGAAGTCA | 132 |
|  | Reverse | TCTTCAGGTGTCAGGCGATT |  |
| β-F1-ATPase | Forward | CATGAAGCAGGTGGCAGGTA | 127 |
|  | Reverse | CAGACGAACACCACGACTCA |  |
| NRF1 | Forward | GCCAGTGAGATGAAGAGAAACG | 166 |
|  | Reverse | CTACAGCAGGGACCAAAGTTCAC |  |
| Nox2 | Forward | TGTATCTGTGTGAGAGGCTGGTG | 156 |
|  | Reverse | CGGGACGCTTGACGAAA |  |
| Nox4 | Forward | TGGAACGCACTACCAGGATG | 202 |
|  | Reverse | TTCGGCACAATACAGGCACA |  |
| VEGF-A | Forward | CCTCGGAGCGGAGAAAGCAT | 126 |
|  | Reverse | TGTCACATCTGCAAGTACGTTCG |  |
| MT-CYB | Forward | ATGAAACATTGGAGTAGTCCTACTATTTACC | 149 |
|  | Reverse | CTACGAGGTCTGTTCCGATATAAGG |  |
| 18S rRNA | Forward | GGTAGTGACGAAAAATAACAATACAGGAC | 141 |
|  | Reverse | ATACGCTATTGGAGCTGGAATTACC |  |
| XBP-1 | Forward | GCCTCCCCTTCTTCATCACT | 177 |
|  | Reverse | TTTCTCTGAGGGGCTGGAAG |  |
